# Supplementary material for: Presenilin mutations deregulate mitochondrial Ca2+ homeostasis and metabolic activity causing neurodegeneration in Caenorhabditis elegans
Source: eLife. 2018 Jul 10;7:e33052. doi: 10.7554/eLife.33052 (PMC6075864; doi:10.7554/eLife.33052)
Supplement: Figure 5—figure supplement 1—source data 1. [file elife-33052-fig5-figsupp1-data1.pdf]

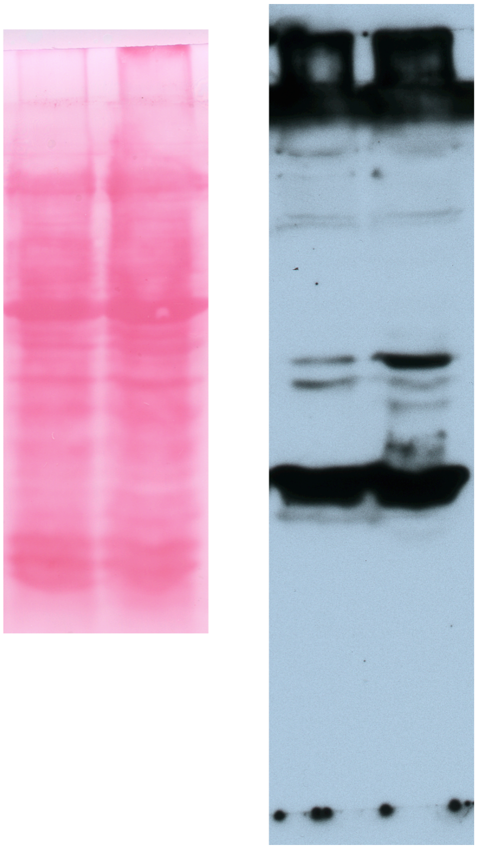

Figure 5-figure supplement 1. Western analysis of pan-neuronal expression of Abeta1-42 in *C. elegans*. Source data.
